# Real-space texture and pole-figure analysis using the 3D pair distribution function on a platinum thin film

**DOI:** 10.1107/S2052252522006674

**Published:** 2022-07-19

**Authors:** Sani Y. Harouna-Mayer, Songsheng Tao, ZiZhou Gong, Martin v. Zimmermann, Dorota Koziej, Ann-Christin Dippel, Simon J. L. Billinge

**Affiliations:** a Center for Hybrid Nanostructures (CHyN), Institute for Nanostructure and Solid-State Physics, University of Hamburg, Hamburg, Germany; b The Hamburg Center for Ultrafast Imaging, Hamburg, Germany; c Department of Applied Physics and Applied Mathematics, Columbia University, New York, USA; d Deutsches Elektronen-Synchrotron DESY, Hamburg, Germany; e Condensed Matter Physics and Materials Science Department, Brookhaven National Laboratory, Upton, NY 11973, USA; ESRF, France

**Keywords:** materials modeling, nanostructure, texture, 3D pair distribution functions, real-space pole figures, real-space fiber plots, *FouriGUI*

## Abstract

An approach is described for studying texture in nanostructured materials. The approach implements the real-space texture pair distribution function (PDF), txPDF, laid out by Gong & Billinge {(2018 ▸). arXiv:1805.10342 [cond-mat]}. It is demonstrated on a fiber-textured polycrystalline Pt thin film. The approach uses 3D PDF methods to reconstruct the orientation distribution function of the powder crystallites from a set of diffraction patterns, taken at different tilt angles of the substrate with respect to the incident beam, directly from the 3D PDF of the sample. A real-space equivalent of the reciprocal-space pole figure is defined in terms of interatomic vectors in the PDF and computed for various interatomic vectors in the Pt film. Furthermore, it is shown how a valid isotropic PDF may be obtained from a weighted average over the tilt series, including the measurement conditions for the best approximant to the isotropic PDF from a single exposure, which for the case of the fiber-textured film was in a nearly grazing incidence orientation of ∼10°. Finally, an open-source Python software package, *FouriGUI*, is described that may be used to help in studies of texture from 3D reciprocal-space data, and indeed for Fourier transforming and visualizing 3D PDF data in general.

## Abstract 2

An approach is described to study texture in nanostructured materials by utilizing the 3D pair distribution function. It is demonstrated how to measure, process and analyze data to obtain the textured 3D pair distribution function on a fiber-textured platinum thin film.

## 1. Introduction

Atomic pair distribution function (PDF) analysis of X-ray, neutron or electron diffraction data is a powerful method to investigate local structure in materials, which can range from amorphous to crystalline (Egami & Billinge, 2012 ▸). It is particularly useful for studying structure in small nanoparticles (Yang *et al.*, 2013 ▸; Grote *et al.*, 2021 ▸), battery materials (Shan *et al.*, 2019 ▸; Hu *et al.*, 2021 ▸) and thin films (Dippel *et al.*, 2020 ▸; Song *et al.*, 2021 ▸).

Conventionally, the PDF assumes the sample to be structurally isotropic, for example, a perfect powder sample. In general, polycrystalline samples may not always conform to this ideal and have preferred orientation of their grains called a crystallographic texture. Historically, depending on the extent of the texture, PDF analysis of such samples is not possible or requires tedious sample preparation, which results in a loss of all texture information (Dippel *et al.*, 2019 ▸; Roelsgaard *et al.*, 2019 ▸; Guo *et al.*, 2013 ▸).

Recently, it was shown how to propagate texture information from samples through the Fourier transform to obtain a 3D PDF of a textured polycrystalline sample, henceforth referred to as a txPDF (Gong & Billinge, 2018 ▸). This would allow, in principle, the determination of orientation distribution functions that encode the texture directly in real space, which may be favorable if the powder grains are nanocrystalline. In the case of weakly textured samples, equations have also been derived to correct the Debye scattering equation for texture information (Cervellino & Frison, 2020 ▸).

In this article, we explore how to extract texture and structural information, in practice, from the txPDF. After reviewing the mathematical framework of the txPDF (Gong & Billinge, 2018 ▸), we describe data acquisition, data processing, texture- and structure-analysis procedures on the example of a fiber-textured Pt thin film. We show how to obtain real-space pole figures and fiber plots, and show how they can be used to obtain quantitative information about the texture in the sample. We also explore how conventional untextured PDFs may be recovered from the data for structural modeling. The experiments are quick, especially at a high-power synchrotron, and the analysis is straightforward. For textured nanostructure, where analysis of well defined Bragg peaks is problematic, this is likely to be a valuable tool for studying texture, and also shows an approach to mitigate texture when the structure of a nanostructured sample is the target.

## 2. Summary of the txPDF method

Here, we summarize the theory of the 3D PDF of textured polycrystalline samples, txPDF, that was introduced by Gong & Billinge (2018 ▸). In contrast to the txPDF, the conventional 1D PDF is herein referred to as pwPDF to differentiate it.

The conventional 1D PDF, pwPDF, from an isotropic powder, *G*(*r*), is obtained by a sine Fourier transform of the reduced structure function, *F*(*Q*) = *Q*[*S*(*Q*) − 1], where the structure function, *S*(*Q*), is related to the measured coherent-scattering intensity, *I*(*Q*), by

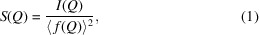

where *Q* is the magnitude of the scattering vector (Egami & Billinge, 2012 ▸). Here, *f*(*Q*) is the atomic form factor and the angle brackets indicate an average over all the chemical species present. The PDF is then obtained via 

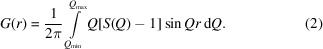

Below we summarize the extension to this equation for the case where the scattering is not isotropic.

pwPDF measurements are often carried out at synchrotron sources due to the abundance of high-energy X-rays. Data are acquired on 2D area detectors. To yield *S*(*Q*), the 2D detector image is azimuthally integrated after applying any masks for problematic pixels in the image, resulting in a 1D function of X-ray intensity versus *Q*. The conversion from detector units to *Q* is carried out with the help of a calibration measurement using a known material. Parasitic scattering is removed, such as measured background signals, and other effects such as multiple scattering and incoherent scattering are also removed, and the intensity is divided by the average of the atomic form factor for the material resulting in *S*(*Q*), which is then propagated through the Fourier transform to obtain the pwPDF. More details may be found in the work of Egami & Billinge (2012 ▸).

**Figure 1:**
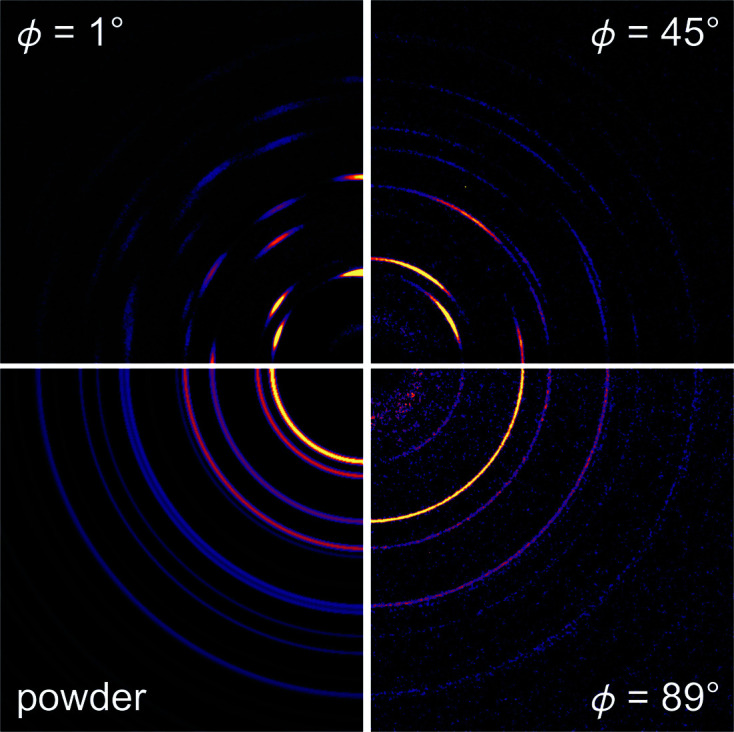
Sections of detector images of the textured Pt sample at different tilt angles ϕ, and a simulated perfect powder Pt detector image. The colorscale is in arbitrary units.

In the case of a textured sample, Bragg peak intensities are not uniform in space. The intensities are increased or decreased relative to the average value depending on the orientation of the sample and the direction in space that the intensity is measured. In the case where the Bragg intensities are measured with a 2D detector, this can result in the diffraction image exhibiting intensity variations around the Debye–Scherrer rings, or sometimes uniform rings but incorrect relative intensities of peaks as a function of *Q*. Fig. 1 ▸ compares detector images of the textured Pt thin-film diffraction pattern at different tilt angles, ϕ, and with an untextured film. Here ϕ is the angle between the incident beam and the plane of the film, and an untextured perfect powder detector image.

**Figure 2:**
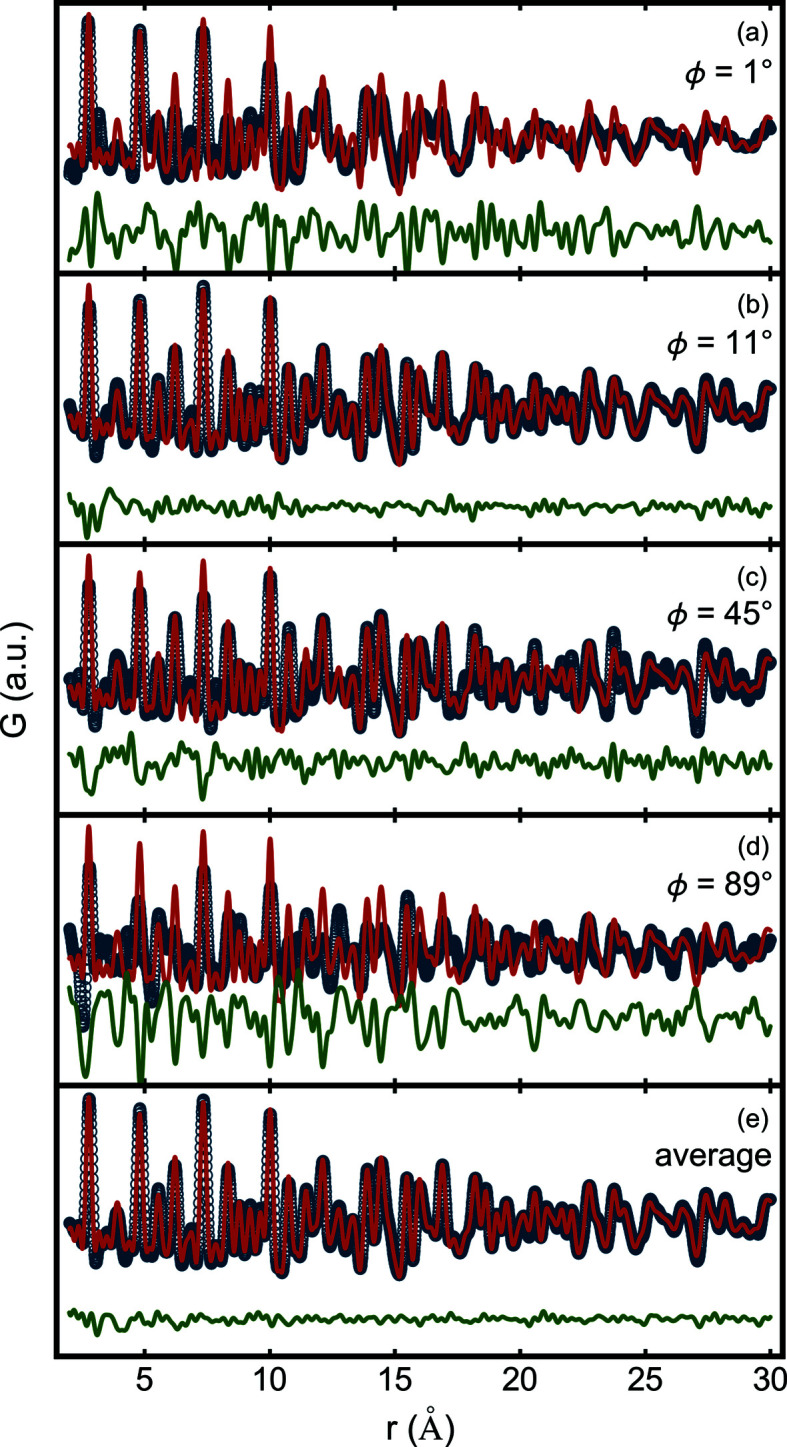
Comparison of measured and best-fit calculated PDFs for diffraction images taken at different sample orientations. The blue circles are the experimental PDFs for sample tilt angles ϕ = 1, 11, 45 and 89°, and from the average of the diffraction images from 1 to 89°. The red curves are the best-fit calculated PDFs from an untextured face-centred cubic model. The green curves are the difference between the calculated and measured PDFs. The PDF refinement from the angle-averaged pattern yields a good result with *R*_*w*_ = 13.5%. Among the single-angle PDFs, ϕ = 11° shows the best refinement result (*R*_*w*_ = 18.1%) and ϕ = 89° shows the worst result (*R*_*w*_ = 63.6%) (Fig. S5).

As a result, the intensity of the peaks in the azimuthally integrated, *I*(*Q*), are different to the *I*(*Q*) of a perfect powder sample, though the peak positions are not altered. If this 1D pattern is handled as if it were from a perfect powder it results in a signal that is distorted from the pwPDF (Fig. 2 ▸). This makes, depending on the extent of the texture, modeling of such pwPDFs impossible (Fig. 2 ▸).

The 3D PDF (Egami & Billinge, 2012 ▸) is a 3D histogram of atomic pair correlations separated by vector **r**, rather than by distance *r*. Previously, the 3D PDF has been used to study diffuse scattering in single crystals (Weber & Simonov, 2012 ▸; Krogstad *et al.*, 2020 ▸). Similar to the case of the pwPDF, the 3D PDF of textured polycrystalline materials, txPDF, may be obtained by propagating the 3D total scattering structure function *S*(**Q**) through the Fourier transform, which is now a 3D transform, 

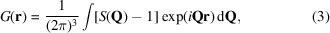

where 

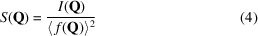

is the 3D scattering function. *S*(**Q**) can be determined from a 3D scattering volume, *I*(**Q**), which can be reconstructed from a set of diffraction images.

Since a diffraction image measured on a 2D area detector is a slice on the surface of the Ewald sphere through reciprocal space, it is possible to reconstruct a 3D reciprocal scattering volume, *I*(**Q**), from a sufficiently complete set of diffraction images with the sample at different orientations, and from there obtain the 3D PDF according to equation (3).

The txPDF formalism describes the texture by introducing the orientation distribution function (ODF) to a polycrystalline 3D PDF (Gong & Billinge, 2018 ▸). The ODF gives the probability density for a crystallite to face orientation Ω, which is the three angles containing the Euler angles of the crystallite in the sample reference frame.

To yield the 3D PDF of a textured polycrystalline sample, txPDF *G*_p_(**r**), a textured polycrystalline 3D structure function *S*_p_(**Q**) is first defined (Gong & Billinge, 2018 ▸) by integrating over all single-crystallite structure functions *S*′(**Q**, Ω) at different orientation angles Ω, weighted by the ODF *D*(Ω), 

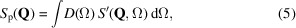

where 

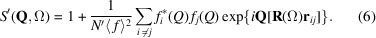

Here *N*′ is the number of atoms in the crystallite, **r**_*ij*_ is the vector from atom *i* to atom *j*, **R**(Ω) is the rotation matrix rotating **r**_*ij*_ from the sample reference frame to the respective crystallite orientation, and *f*_*i*_ and *f*_*j*_ are the atomic form factors of atom *i* and atom *j*, respectively, in the crystallite. These equations are derived more fully by Gong & Billinge (2018 ▸).

The txPDF is then obtained by propagating *S*_p_(**Q**) through the Fourier transform, 

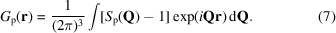




## 3. Example: a nanostructured thin film with fiber texture

**Figure 3:**
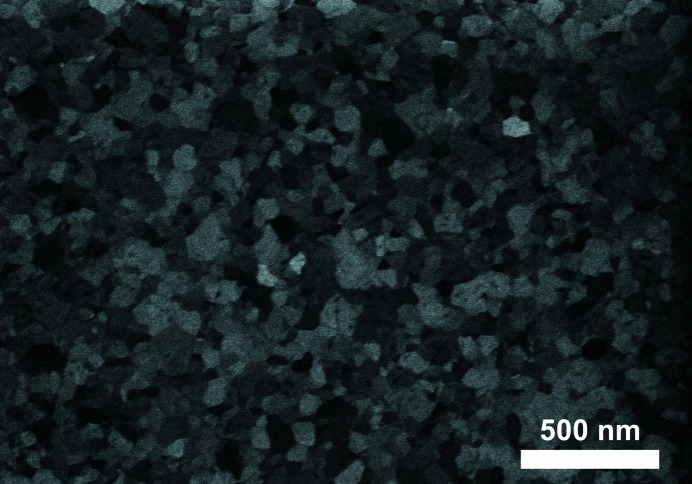
An SEM image of the Pt thin-film surface showing a polydisperse size distribution and different shapes of the Pt grains in the lateral direction.

Here we test the txPDF method on experimental data for the case of a nanostructured polycrystalline Pt thin film that exhibits a strong fiber texture with 〈111〉 crystallographic directions tending to align perpendicular to the film direction. The sample was fabricated by sputter deposition (CS 500 ES, Von Ardenne GmbH) of a 50 nm Pt layer (target from FHR Anlagenbau GmbH) on a glass substrate of size 1 × 1 cm with an intermediate evaporated 15 nm aluminium oxide adhesion layer (granulate from Evochem GmbH). A scanning electron microscopy (SEM) image of the film is shown in Fig. 3 ▸.

**Figure 4:**
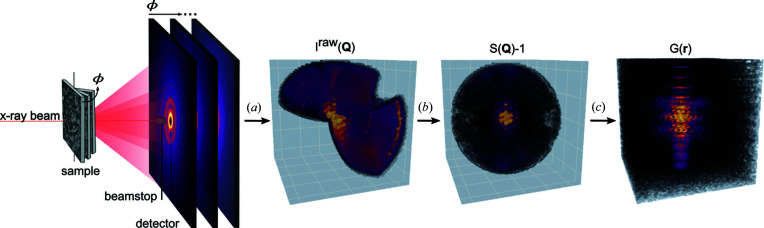
The experimental and data-processing procedure. (*a*) Reconstruction of a tilt series of 2D scattering images to a 3D scattering volume *I*(**Q**). (*b*) Processing to the total scattering structure function *S*(**Q**), with application of symmetry averaging, interpolation, and cutoff beyond **Q**_min_ and **Q**_max_. (*c*) Fourier transformation to the txPDF *G*(**r**).

Fig. 4 ▸ summarizes the experimental procedure and data processing. The measurement was performed at beamline P07 at PETRA III, Deutsches Elektronen-Synchrotron DESY, Hamburg, Germany (Gustafson *et al.*, 2014 ▸). The beam energy was 79.5 keV. The beam size was 0.5 × 0.5 mm. Data were collected on a PerkinElmer XRD1621 detector. The detector was calibrated by diffraction images of a lanthanum hexaboride coated glass substrate using the *pyFAI* software (Ashiotis *et al.*, 2015 ▸).

Diffraction images were measured at angles from ϕ = 1 to 89° with a step size of 1°. The set of diffraction images was transformed from detector coordinates to reciprocal-space coordinates using the Python library *meerkat* (Simonov, 2019 ▸). *Meerkat* loads the diffraction images and bins each pixel to points in reciprocal space to reconstruct the 3D scattering volume.

Next, it was necessary to make corrections to the data to convert raw intensities to the properly corrected and normalized *S*(**Q**) function that can be Fourier transformed to the txPDF. The measured scattering intensity from a sample will change during the measurement due to various experimental reasons, *e.g.* the illuminated volume of the sample at different tilt angles and changes in incident-beam intensity, and this needs to be normalized. If the geometry of the sample is well known and the incident beam monitored, such effects on the intensity can be explicitly corrected (Egami & Billinge, 2012 ▸). In this experiment, the main intensity variation originates from the varying tilt angle. In general, to account for such variations, we can apply a *post hoc* correction for these effects by applying a systematic scaling to the diffraction pattern as a whole. To determine the *post hoc* scaling factor we did the following. First, we assumed that away from the *Q* values where Pt Bragg reflections exist, the scattering intensity is coming predominantly from the glass substrate and other incoherent sources (such as Compton scattering and any fluorescence), all of which are isotropic and should scale with the illuminated sample volume. In this case, the scaling can be obtained from the total sum of the intensity in the azimuthally averaged *I*′(*Q*), *i.e.* 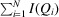 , where the prime denotes that this *I*′(*Q*) excludes regions that contain Pt Bragg reflection intensities, and the sum is over *N* intensity bins. This *post hoc* correction will be difficult to execute whenever the Bragg reflections are not easily distinguishable from the background scattering, such as in the case of low-symmetry or multiphase systems with too many Bragg reflections, or very small nanostructures where the Bragg reflections are too broad. In such cases, it is favorable to apply scaling based on simple geometrical considerations (Egami & Billinge, 2012 ▸). The value of the applied scale factor versus ϕ is shown in Fig. S2 of the supporting information . It is compared with the 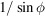 scaling that is expected for our sample geometry (Egami & Billinge, 2012 ▸). The agreement is very good, except in the region of glancing angle, where the 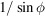 diverges but the measured intensity from the sample turns over and comes back down. This could be because of scattering from the edges of the sample, imperfect alignment of the sample and the detector, or due to the beam footprint exceeding the size of the sample. If the sample is even a tiny bit too high or too low at grazing incidence, the beam can partially miss the sample or be predominantly absorbed by the substrate, respectively, which would cause the observed effect. These factors make grazing-incidence experiments rather specialized in general (Feidenhans’l, 1989 ▸; Dosch, 1992 ▸; Gustafson *et al.*, 2014 ▸). Here, we are not concerned about making a high-quality grazing-incidence PDF measurement, but rather measuring the film over a wide angular range, and the *post hoc* correction allows us to make use of the scattering deeper into the grazing-incidence regime, even with a non-ideal grazing-incidence alignment and setup there.

**Figure 5:**
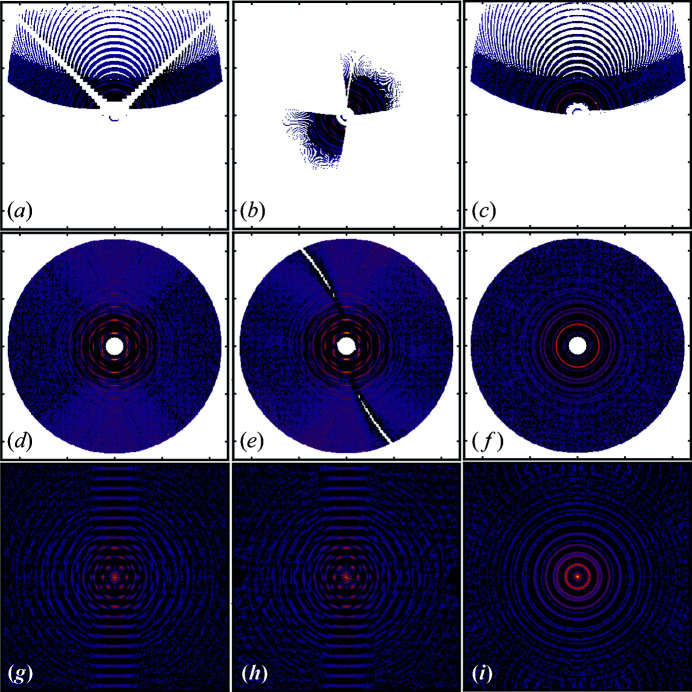
Slices through (*a*)–(*c*) the raw reconstructed scattering volume, (*d*)–(*f*) the processed total scattering structure function and (*g*)–(*i*) the txPDF, with arbitrary units on a logarithmic scale. The slices in the first column are perpendicular to *x*_s_, in the second column are perpendicular to *y*_s_ and in the third column are perpendicular to *z*_s_, where *x*_s_, *y*_s_ and *z*_s_ are the sample coordinates, see the main text for details. All slices are through the origin of the diffraction space. The edge length is from −23.9 to 23.9 Å^−1^ in (*a*)–(*f*) and from −26.3 to 26.3 Å in (*g*)–(*i*), respectively. The void features in (*a*)–(*c*) result from the relatively high tilt step size of the measurement. The straight void lines in (*a*) result from masked high-absorption features from the substrate. The void lines in (*b*) and (*e*) are due to the masking of the beamstop.

Representative slices through reciprocal space of the normalized transformed intensity data are shown in Figs. 5 ▸(*a*)–5 ▸(*c*).

There are large regions of reciprocal space missing. This is because of the rather small tilt range and relatively high tilt step size in our experiment. This can be remedied in general by taking many more images on a finer tilt grid (for example, steps of 0.1° rather than the 1° steps used here), measuring over a wider angular range, and ultimately doing this around two perpendicular rotation axes. However, in the current case, the high symmetry of the structure and the texture allowed us to reconstruct the reciprocal space rather completely, even from this limited set of data, through symmetrization. The symmetrization is discussed below. Finally, the data were interpolated to recover small missing regions within the scattering volume that were still present after symmetrization. The code we wrote to do the symmetrization can be found at https://github.com/diffpy/fourigui/tree/main/further_processing_code.

We define the film plane normal to be the sample-*z* direction, *z*_s_, and select two perpendicular directions in the plane of the film to be the sample-*x* direction, *x*_s_, and the sample-*y* direction, *y*_s_. We expect that, referenced to these sample directions, the fiber texture dictates that the scattering will look the same viewed along the *x*_s_ and *y*_s_ directions, and look different, but circularly symmetric, when viewed along *z*_s_. If we take the actual diffraction images and reconstruct them into this 3D diffraction space, and make slices through the reconstructed diffraction space perpendicular to *x*_s_, *y*_s_ and *z*_s_, going through the center of the reciprocal space, we get the images in Figs. 5 ▸(*a*)–5 ▸(*f*). It is difficult to verify the predictions in the unsymmetrized data [Figs. 5 ▸(*a*)–5 ▸(*c*)], but is very apparent after filling in the missing regions [Figs. 5 ▸(*d*)–5 ▸(*f*)]. Due to the strong fiber texture, our dataset exhibits rotational symmetry about the fiber axis or *z*_s_. Hence, we can readily apply all symmetry operations about the 111 fiber axis, which can be derived from the body-centred cubic reciprocal crystal structure of Pt (Table S3 of the supporting information). The symmetrization consists of individually applying the symmetry operations to the reconstructed scattering volume and eventually averaging the symmetry-operated datasets.

The symmetrized diffraction signal is then converted to *S*(**Q**) − 1 by dividing by the average-form-factor squared and rescaling, as shown in Fig. 4 ▸. In practice, the measured intensity comprises several features in addition to the coherent-scattering intensity, such as incoherent Compton scattering, multiple scattering, self absorption and polarization effects of the X-ray beam, which we should correct for before Fourier transforming to the PDF (Egami & Billinge, 2012 ▸). In the case of the pwPDF, the scattering intensity can be corrected by modeling a polynomial correction term β(*Q*) (Juhás *et al.*, 2013 ▸). By assuming that the scattering contributions are isotropic, we can calculate β(*Q*) by propagating the spherical integration of *S*(**Q**) − 1, *S*(*Q*) − 1, which should resemble the powder average, through the *PDFgetX3* algorithm (Juhás *et al.*, 2013 ▸). We then isotropically subtract the polynomial correction β(**Q**) from *S*(**Q**) − 1 to retrieve the coherent-scattering fraction. The correction procedure is schematically represented in Fig. S7. The same slices as shown in Figs. 5 ▸(*a*)–5 ▸(*c*), symmetrized and in the form of *S*(**Q**) − 1, are shown in Figs. 5 ▸(*d*)–5 ▸(*f*). The 3D diffraction intensities from a perfect powder would appear as concentric spheres in this reconstructed scattering volume, and so slices through the volume that go through the origin of reciprocal space, as we plotted in the figure, would show uniform circles. This is indeed what we see in the slice looking down *z*_s_, the fiber axis [Fig. 5 ▸(*f*)]. The circles are also evident in the slices that are perpendicular, *x*_s_ and *y*_s_ [Figs. 5 ▸(*d*) and 5 ▸(*e*)]. However, the circles appear broken and have non-uniform intensities as you go around the azimuth, as a result of the texture. The two slices are, of course, identical to each other due to the fiber symmetry and the resulting symmetrization step.

The scattering volume is further interpolated and intensity values beyond *Q*_min_ and *Q*_max_ are set to zero before carrying out the 3D Fourier transformation to obtain the txPDF *G*(**r**) [equation (3)], as shown in Fig. 4 ▸, and the slices are shown in Figs. 5 ▸(*g*)–5 ▸(*i*). The spherical symmetry of the scattering from a perfect powder also results in a spherically symmetric *G*(**r**). In the false-color images of Figs. 5 ▸(*g*)–5 ▸(*i*) we would expect to again see circular patterns of increased and reduced *G* values. These circles are evident when viewed down the *z*_s_ axis [Fig. 5 ▸(*i*)], but, as before, become non-uniform around the azimuth when viewed along the *x*_s_ and *y*_s_ axes [Figs. 5 ▸(*g*) and 5 ▸(*h*)].

## 4. Texture analysis

The reconstructed reciprocal space and resulting real-space txPDF, presented above, contain concentric spheres of intensity at values of reciprocal-lattice vector magnitude, |**H** ^*hkl*^| (reciprocal space), and interatomic vector magnitude, |**r** ^*i*,*j*^| (real-space txPDF), that become broken up and non-uniform due to the presence of texture. There is, overall, a resemblance in the patterns of intensity between *G*(**r**) and those seen in reciprocal space, as they contain the same fiber-texture information, but peaks in *G*(**r**) appear at positions corresponding to interatomic vectors rather than reciprocal-lattice vectors. We now consider how to extract texture information from *G*(**r**).

**Figure 6:**
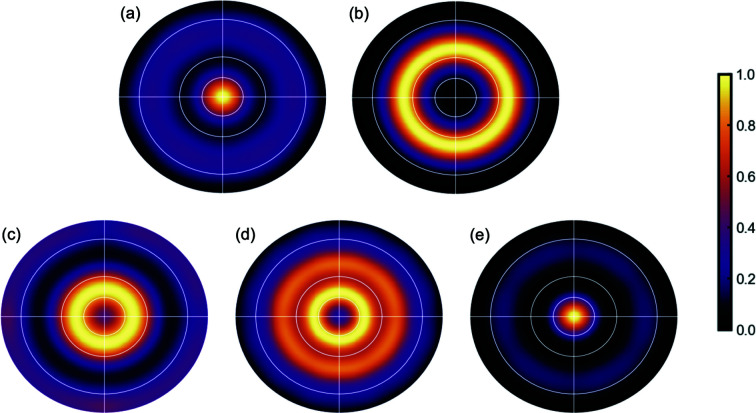
Reciprocal-space pole figures derived from the scattering volume *S*(**Q**). Peaks: (*a*) {111}, (*b*) {002}, (*c*) {022}, (*d*) {133} and (*e*) {222}.

One way to understand the crystalline texture is to use the pole-figure construction (Bunge, 1982 ▸). In this construction we consider a reciprocal-lattice vector from a single crystal grain, 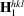 , and place its tail at the origin of our diffraction space. We then consider this same reciprocal-lattice vector from every crystal grain, *m*, in the sample and do the same. This results in a construction where the tips of all the reciprocal-lattice vectors, 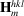 , lie on the surface of a sphere of radius |**H** ^*hkl*^|, which we here refer to as the reciprocal-space pole sphere. In a perfect powder there would be a uniform density of vector tips over the entire surface of the reciprocal pole sphere. When texture is present, some regions of the surface of the reciprocal-space pole sphere are covered more densely than others. In the case of a fiber texture such as ours, it is conventional to align the north pole of the reciprocal-space pole sphere with the sample fiber axis. In this case we find high densities of pole vectors that form spherical segments (or spherical caps for the 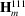 vector) on the pole sphere. The pole figure is created by taking a stereographic projection of the vector tips on the reciprocal-space pole sphere. In practice, for a given set of reciprocal-lattice vectors, 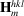 , the intensity of scattering on a spherical annulus centered at *Q* = |**H** ^*hkl*^| in the reconstructed diffraction space is proportional to this quantity (assuming there are no other overlapping reciprocal-lattice vectors). The stereographic projection of these intensities results in the pole figure for that particular reciprocal-lattice vector, or set of symmetry-equivalent reciprocal-lattice vectors. In the pole figure, the fiber texture appears as circular rings of bright intensity, as shown in Fig. 6 ▸.

**Figure 7:**
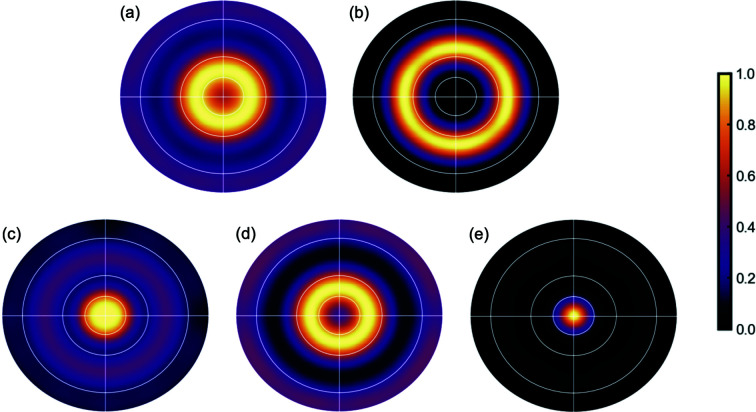
Real-space pole figures derived from the txPDF *G*(**r**). Peaks: (*a*) 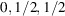 , (*b*) 0, 0, 1, (*c*) 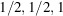 , (*d*) 0, 1, 1 and (*e*) 1, 1, 1.

The standard pole-figure construction is inaccurate for materials that are not long range ordered since the scattering is not concentrated in sharp Bragg peaks lying at the reciprocal-lattice points. However, as shown above, it is possible to describe texture in terms of real-space structural vectors through the 3D txPDF construction. We can therefore explore the real-space analog of the pole figure as a stereographic projection of the intensity on the surface of the real-space pole sphere, which is defined analogously to the reciprocal-space pole sphere but for interatomic vectors **r** ^*i*,*j*^. We can call the resulting stereograms: ‘real-space pole figures’. Projecting on the equatorial plane perpendicular to the fiber axis *z*_s_ yields, as in the reciprocal-space pole figure, uniform concentric rings of bright intensity. However, despite the similar appearance of the reciprocal- and real-space pole figures, they are not equivalent texture representations as they come from very different origins, being constructed in different spaces. They encode the same information but in different ways, so direct visual comparison between them can be problematic. Representative real-space pole figures from our sample are shown in Fig. 7 ▸.

**Figure 8:**
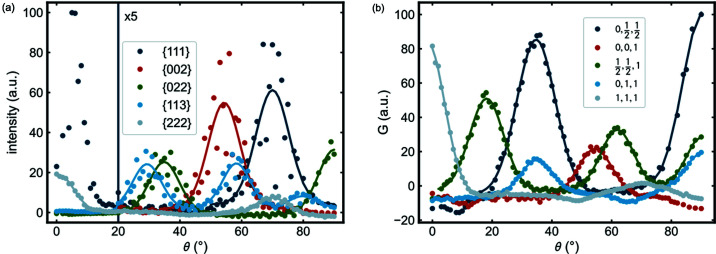
(*a*) Reciprocal-space fiber plots of the first five X-ray diffraction reflections. (*b*) Real-space fiber plots of the first five PDF peaks. Gaussian fits of the peaks in the fiber plots are shown as solid traces. The fit of the reciprocal 111 reflection at θ = 0° is omitted because of low data quality at that region and poor resolution at low *Q* after the conversion to polar coordinates. The fit of the real 111 peak at θ = 70.53° is omitted because of a poor fit result due to the low intensity. The intensities of the reciprocal- and real-space fiber plots were retrieved by summing over all intensity values at the *H* ^*hkl*^ value of the respective peak ± 0.1 Å^−1^ from the scattering volume *S*(**Q**) and over the *r* ^*ij*^ values of the respective interatomic distance ± 0.1 Å from the txPDF *G*(**r**), respectively. Increasing the integration width for the fiber-plot construction does not reduce the high noise in the reciprocal-space fiber plot. All intensities beyond the vertical line at θ = 20° in (*a*) are multiplied by 5 since intensity values at high θ are hardly visible if put on the same scale as the high intensities at low θ values.

Due to the fiber texture and the choice of projection plane, the pole-figure intensities are independent of the azimuthal angle and we can integrate them azimuthally to obtain 1D plots called fiber plots (Bunge, 1982 ▸; He, 2018 ▸). In the fiber-plot representation the intensity is plotted against the zenith (*i.e.* polar) angle, θ, which is the angle between the position vector of the pixel on the real/diffraction-space spheres and the polar axis, *z*_s_. In the center of the pole figure θ is 0°, and the outer ring at the edge of the pole figure is at θ = 90°. The reciprocal- and real-space fiber plots corresponding to these pole figures are shown in Fig. 8 ▸.

Assuming the fiber texture gives a Gaussian distribution of poles as a function of the zenith angle θ, we can fit a series of Gaussian functions to the peaks in the fiber plots. Apart from some noise in the data, we get stable fits. The positions of the fitted peaks in the reciprocal- as well as the real-space fiber plots, 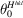 and 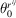 , respectively, are in good agreement with the expected angle between the respective reciprocal/real vector and the 111 axis, which is the fiber axis (see Tables 1 ▸ and 2 ▸). The width of the peaks in the fiber plots comes from the angular distribution given by the degree/extent of the fiber texture convoluted with the intrinsic width of the peak in the 3D scattering volume or txPDF. We yielded similar results for the full width at half-maximum (FWHM) in the reciprocal and the real fiber plots, which confirms that our treatment of the data to obtain the txPDF is valid. The real FWHMs, at ∼13.6 ± 2.1°, are slightly larger than the reciprocal FWHMs (11.6 ± 1.2°), where the uncertainties represent the spread in values obtained from all the different reciprocal/real vectors. Although the difference between the real and reciprocal measures is within the variation of values, the txPDF is giving a width for the fiber texture that is rather consistently larger than that obtained from the reciprocal data. We attribute this to the convolution of the angular distribution from the fiber texture with the different intrinsic width of the PDF versus the Bragg peaks. To get a measure of the intrinsic peak widths, we compared the FWHMs of Gaussian fits of the first three peaks in the spherical integration of the scattering volume and the txPDF (Fig. S4). The PDF peak FWHMs (0.42 ± 0.02 Å) are slightly larger than the Bragg peak FWHMs (0.32 ± 0.04 Å^−1^), which is consistent with the above statement. The uncertainties represent the spread in values obtained from the different fitted peaks. Furthermore, a possible mosaicity in the crystals could contribute to the broader real-space fiber-plot peaks. The presence of mosaicity would be seen as a distribution in interatomic vector angles within a single crystallite, and seen in the txPDF but not in the reciprocal-space pole figures and fiber plots. We also note from Fig. 8 ▸ that the scatter of the data points is much greater in the reciprocal-space fiber plots than the real-space fiber plots. We are uncertain as to the origin of this effect, but for the real-space plots it means that the variation of FWHM values is larger than any variation due to the noise in the data.

Although, in the case of the fiber texture, pole figures and fiber plots yield a straightforward and rather precise picture of the texture, in general the texture of a sample cannot be comprehensively described by the two angles of a pole figure. The more complete texture representation is the ODF, *D*(Ω), which describes the crystallite orientation by the three Euler angles in a density probability function (Bunge, 1982 ▸). The ODF can be evaluated from a series of pole figures, where the number needed depends on the symmetry of the texture and of the crystal (Bunge, 1982 ▸). However, in general, the ODF may be reconstructed directly by modeling the 3D structure function *S*(**Q**) or txPDF *G*(**r**) (Gong & Billinge, 2018 ▸). If the structure function of the reference crystallite *S*′(**Q**) [equation (6)] is known, as is often the case, we can rotate *S*′(**Q**) at different orientations of **Q** and add the contributions weighted by an initial assumption of the ODF to build the polycrystalline structure function *S*_p_(**Q**) [equation (5)]. Then we can fit *S*_p_(**Q**) to the measured *S*(**Q**) by modeling the ODF in a regression loop. Equivalently, the ODF can be retrieved by a similar modeling of the txPDF *G*(**r**) [equation (7)] (Gong & Billinge, 2018 ▸).

## 5. Structural analysis

Here we discuss extracting meaningful structural information from PDFs that were measured from textured samples. This is relevant for the case where the experimenter is not trying to characterize the sample texture *per se* but is interested in the local structure of a textured sample. In favorable circumstances, texture-related intensity changes in Bragg peaks can be modeled in a Rietveld refinement (Gilmore *et al.*, 2019 ▸). This is less well developed in real-space refinements, though for weak textures, a model has been proposed (Cervellino & Frison, 2020 ▸). Here we explore whether sufficiently texture-free PDFs, which can then be refined in existing PDF modeling programs such as *PDFgui* (Farrow *et al.*, 2007 ▸), can be obtained by averaging data collected at different sample angles. As a test, we will use the dataset from the fiber-textured Pt thin film as an example.

In theory, measuring the diffraction pattern by sampling every orientation of the sample with equal statistics will result in a spherically homogeneous diffraction pattern that can be processed as an ideal powder pattern. Using a 2D detector and rotating 180° about two mutually perpendicular axes perpendicular to the beam can accomplish this for any crystallographic and texture symmetry, if correct counting statistics are ensured. Correct counting statistics will depend on flux and exposure-time weighting, but also on any geometrical and absorption effects that result in changes in illuminated sample volume. These can be readily controlled for simple sample shapes (spherical, cylindrical, planar/film, *etc*.), as we describe below. In many cases, such as the case of the fiber-textured sample, the averaging may be accomplished by rotating about a single axis perpendicular to the beam, provided that the fiber axis doesn’t lie along the rotation axis. For experimental setups where this is possible, and where data rates are sufficiently high, it probably represents the best approach. In our case, the fiber texture resulted from oriented growth of the polycrystalline film on the substrate. In this case, the illuminated volume is well described by the area of the beam footprint on the sample, which is given by the beam width, *w*, times 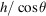 , where *h* is the beam height and θ is the angle the substrate plane normal makes with the incident beam. Since *w* and *h* don’t vary with incident angle, we can determine that the measured intensities should be multiplied by 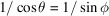 (Egami & Billinge, 2012 ▸), where ϕ = π/2 − θ is the grazing angle the incident beam makes with the substrate. For even higher accuracy, we can use the same normalization/scaling approach that is applied to the diffraction images before reconstructing to the 3D scattering volume, as discussed in the experimental section.

Fig. 2 ▸(*e*) shows a fit of an untextured model to the PDF that was obtained by a properly weighted average of the PDFs from all sample angles collected in our dataset. Indeed, the fit is superior to the fits at any fixed sample angle, as evident from the difference curve. Table 3 ▸ shows the refined structural parameters and the goodness of fit parameter, *R*_*w*_, of a few single-angle PDF refinements and the averaged dataset.

Indeed, the best fit of the untextured model to the data is obtained for averaged data (*R*_*w*_ = 13.5%) compared with any single-fixed-angle dataset. The best agreement between the powder average model and a single-angle dataset is achieved for ϕ = 11° with *R*_*w*_ = 18.1%. For this powder texture the agreement gets better as the incident angle gets shallower, though at small incident angles (below ϕ = 11°) the measured PDFs seem to become less reliable. The refined parameters of the fit to the ϕ = 11° are also closer to those obtained for the averaged data than for the other angles. A plot of the best-fit *R*_*w*_ versus ϕ is shown in Fig. S5, showing this behavior in a more quantitative fashion. Based on this analysis, when looking at data from a textured thin-film sample, the best strategy for obtaining PDFs that represent a texture-less structure and are suitable for conventional quantitative structural modeling in real space is to rotate the sample ±89° about an axis perpendicular to the beam (from a starting position with the film perpendicular to the beam) and to average the data as described in this article. If this is not possible or proves inconvenient, for similarly textured films (*i.e.* fiber textured perpendicular to the film plane), taking a single dataset at an incident angle of ∼10° grazing angle to the film will give a reasonably untextured signal.

Beyond summing raw images from different angles, it is also possible to obtain an 1D powder pattern and PDF resembling that of an untextured sample by integrating spherical shells in the reconstructed txPDF. In theory, independent of the kind, extent and orientation of the texture, this integration should resemble the precise isotropic powder average and can be processed as such. Correspondingly, a similar integration of the reconstructed scattering volume can be analyzed as an isotropic powder pattern. We were not able to test this in the current case because our test data were not collected on a fine-enough grid of angles to avoid interpolation errors that introduced significant aberrations. We have not investigated the experimental conditions to obtain such an isotropic or de-textured PDF, but a dataset with 0.1° step size and a total tilt range of 90° along two perpendicular axes, *i.e.* 180°, is sufficient for high-quality reconstructions of reciprocal space in single-crystal diffuse-scattering measurements, and would undoubtedly work in this context too. This would add up to 1800 diffraction images, which can be acquired in ∼1–2 h at a synchrotron, assuming a few seconds of acquisition time per diffraction image.

Finally, the txPDF can also be applied to study other anisotropic structural properties such as strain and stress by analyzing the txPDF along different directions in a small angular range. A txPDF of an untextured but strained or stressed sample would show peaks that appear as concentric spherical shells that are non-uniform, *i.e.* they exhibit varying peak shapes and radii at different angular directions. A dataset to study stress or strain requires a measurement over a large tilt range, since symmetrization procedures, which could recover void regions in the reconstructed scattering volume, result in a loss of strain and stress information.

## 6. Software

The software used to carry out the work in this article has been turned into a Python package and is being released under an open-source license. The package is called *FouriGUI* and is available through the DiffPy website (https://diffpy.org). It is available on the Python package index and on Anaconda through the conda-forge channel. The code is hosted on GitHub at https://github.com/diffpy/fourigui.

*FouriGUI* loads a 3D total scattering volume, for example, obtained from a rotation image set, using a software package such as *Xcavate* (Estermann & Steurer, 1998 ▸), *XDS* (Kabsch, 2010 ▸) or *meerkat* (Simonov, 2019 ▸), and performs the fast Fourier transform to the txPDF with optional cutoff frequencies. It also incorporates visualization capabilities for viewing the data in real and reciprocal space. It always displays one slice perpendicular to one of the three axes of either the scattering volume or the txPDF obtained with or without cutoff frequencies and allows easy scrolling through the full volume to visualize the intensity distribution. Fig. S6 provides further insight about the utilization of *FouriGUI*.

## 7. Conclusions

Here we have presented a practical implementation of the 3D PDF method of textured polycrystalline samples, txPDF. We demonstrated the procedure for measuring and analyzing data to obtain the txPDF using a fiber-textured Pt thin-film sample. Firstly, 2D diffraction images of the Pt thin film measured at different tilt angles were reconstructed to a scattering volume in 3D reciprocal space. The scattering volume was then processed to the 3D total scattering structure function and propagated through the 3D Fourier transform to yield the txPDF in real space. Furthermore, we presented a definition and demonstration of real-space pole figures. We also presented *FouriGUI*, a Python package for carrying out the steps in the procedure.

The txPDF method may be used to study texture in highly nanocrystalline samples where Bragg reflections are broad and overlapped preventing the study of texture directly in reciprocal space. Our findings show a strong fiber texture of approximately Gaussian distribution of 〈111〉 plane vectors that is centered on the Pt thin-film sample’s plane normal. The approach we have presented may also be used to obtain approximately isotropic 1D PDFs from textured samples, which will be useful when the structure of a sample is sought, rather than a measure of its texture, but the experimental data are textured. A good isotropic PDF approximation was obtained from the weighted average of the diffraction images, yielding a reasonable PDF refinement, 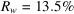 , from the averaged PDF compared with the single-tilt-angle PDFs with *R*_*w*_ values ranging from 18.1 to 63.6%. We also showed that an approximately ideal 1D PDF could be obtained from a not-quite grazing incidence (10° incident angle) single measurement in the case of a thin film with a fiber texture.

The txPDF is very promising to study nanocrystalline thin films, as they are likely to be textured and enable a straightforward measurement of the tilt series. However, the txPDF can be applied in general to materials of any geometry. Furthermore, the methodology of the txPDF can be expanded to study nanoscale materials with different structural anisotropies, such as anisotropic strains. 

## Figures and Tables

**Table 1:** Reciprocal-space fiber-plot peak fit results 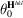
 is the fitted peak position.

Lattice plane	Reference angle to [111] (°)	Gaussian fit result	
		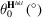	FWHM (°)
{111}	0.0	–	–
	70.5	70.0 (6)	13 (2)
{002}	54.7	54.3 (5)	12.2 (14)
{022}	35.3	34.9 (3)	11.4 (13)
	90.0	89.8 (7)	12 (2)
{113}	29.5	29.3 (3)	10.9 (12)
	58.5	58.3 (3)	10.4 (14)
	80.0	79.8 (6)	10 (3)
{222}	0.0	0.9 (3)	11.4 (6)
	70.5	69.8 (2)	12.5 (7)

**Table 2:** Real-space fiber-plot peak fit results The interatomic vectors from atom *i* to atom *j* are represented in terms of components using a basis of the crystallographic lattice vectors and assuming that the origin is placed on atom *i*. Furthermore, *r* ^*ij*^ is the distance between atom *i* and atom *j*, and 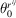 is the fitted peak position.

Position of atom *j*	*r* ^*ij*^ (Å)	Reference angle to [111] (°)	Gaussian fit result	
			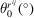	FWHM (°)
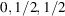	2.77	35.3	34.72 (7)	15.2 (2)
		90.0	90.5 (6)	15.7 (10)
0, 0, 1	3.92	54.7	54.47 (10)	13.5 (3)
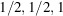	4.81	19.5	18.16 (9)	13.4 (3)
		61.9	61.92 (8)	13.0 (3)
		90.0	90.2 (6)	11.8 (13)
0, 1, 1	5.55	35.3	35.04 (10)	12.8 (3)
		90.0	90.1 (7)	12 (2)
1, 1, 1	6.80	0.0	−1.5 (4)	13.3 (6)
		70.5	–	–

**Table 3:** 1D PDF refinement results for the Pt thin film measured at different incidence angles, ϕ, and a fit to an experimental PDF obtained as an average over all tilt angles Refinement parameters: *a* is the lattice parameter, *U*_iso_ is the isotropic atomic displacement parameter, δ_2_ is the coherent thermal-motion parameter and *R*_*w*_ is the goodness of the fit.

ϕ (°)	*a* (Å)	*U*_iso_ (Å^2^)	δ_2_ (Å^2^)	*R*_*w*_ (%)
1	3.924	0.00879	4.99	42.2
11	3.932	0.00538	3.66	18.1
23	3.929	0.00503	4.25	20.0
45	3.929	0.00548	5.45	27.8
67	3.932	0.00518	6.24	35.7
89	3.933	0.00398	7.80	63.6
Avg	3.931	0.00558	3.90	13.5

## References

[bb1] Ashiotis, G., Deschildre, A., Nawaz, Z., Wright, J. P., Karkoulis, D., Picca, F. E. & Kieffer, J. (2015). *J. Appl. Cryst.* **48**, 510–519. 10.1107/S1600576715004306 PMC4379438 25844080

[bb2] Bunge, H. (1982). *Texture Analysis in Materials Science*. Vol. 1. Oxford: Butterworth-Heinemann

[bb3] Cervellino, A. & Frison, R. (2020). *Acta Cryst.* A**76**, 302–317. 10.1107/S2053273320002521 PMC7233016 32356781

[bb4] Dippel, A.-C., Gutowski, O., Klemeyer, L., Boettger, U., Berg, F., Schneller, T., Hardtdegen, A., Aussen, S., Hoffmann-Eifert, S. & Zimmermann, M. v. (2020). *Nanoscale*, **12**, 13103–13112. 10.1039/d0nr01847c 32543637

[bb5] Dippel, A.-C., Roelsgaard, M., Boettger, U., Schneller, T., Gutowski, O. & Ruett, U. (2019). *IUCrJ*, **6**, 290–298. 10.1107/S2052252519000514 PMC6400183 30867926

[bb6] Dosch, H. (1992). *Critical Phenomena at Surfaces and Interfaces: Evanescent X-ray and Neutron Scattering*. New York: Springer-Verlag.

[bb7] Egami, T. & Billinge, S. (2012). *Underneath the Bragg Peaks: Structural Analysis of Complex Materials*. Vol. 16. Burlington: Elsevier Science.

[bb8] Estermann, M. A. & Steurer, W. (1998). *Phase Transit.* **67**, 165–195.

[bb9] Farrow, C. L., Juhas, P., Liu, J. W., Bryndin, D., Božin, E. S., Bloch, J., Proffen, T. & Billinge, S. J. L. (2007). *J. Phys. Condens. Matter*, **19**, 335219. 10.1088/0953-8984/19/33/335219 21694142

[bb10] Feidenhans’l, R. (1989). *Surf. Sci. Rep.* **10**, 105–188.

[bb11] Gilmore, C. J., Kaduk, J. A. & Schenk, H. (2019). *International Tables for Crystallography*. Vol. H. Chester: International Union of Crystallography.

[bb12] Gong, Z. & Billinge, S. J. L. (2018). arXiv:1805.10342 [cond-mat].

[bb13] Grote, L., Zito, C. A., Frank, K., Dippel, A.-C., Reisbeck, P., Pitala, K., Kvashnina, K. O., Bauters, S., Detlefs, B., Ivashko, O., Pandit, P., Rebber, M., Harouna-Mayer, S. Y., Nickel, B. & Koziej, D. (2021). *Nat. Commun.* **12**, 4429. 10.1038/s41467-021-24557-z PMC8292528 34285227

[bb14] Guo, W., Dmowski, W., Noh, J.-Y., Rack, P., Liaw, P. K. & Egami, T. (2013). *Metall. Mater. Trans. A*, **44**, 1994–1997.

[bb15] Gustafson, J., Shipilin, M., Zhang, C., Stierle, A., Hejral, U., Ruett, U., Gutowski, O., Carlsson, P.-A., Skoglundh, M. & Lundgren, E. (2014). *Science*, **343**, 758–761. 10.1126/science.1246834 24482118

[bb16] He, B. B. (2018). *Two-dimensional X-ray Diffraction*. 2nd ed. Hoboken: John Wiley & Sons.

[bb17] Hu, E., Li, Q., Wang, X., Meng, F., Liu, J., Zhang, J.-N., Page, K., Xu, W., Gu, L., Xiao, R., Li, H., Huang, X., Chen, L., Yang, W., Yu, X. & Yang, X.-Q. (2021). *Joule*, **5**, 720–736.

[bb18] Juhás, P., Davis, T., Farrow, C. L. & Billinge, S. J. L. (2013). *J. Appl. Cryst.* **46**, 560–566.

[bb19] Kabsch, W. (2010). *Acta Cryst.* D**66**, 125–132. 10.1107/S0907444909047337 PMC2815665 20124692

[bb20] Krogstad, M. J., Rosenkranz, S., Wozniak, J. M., Jennings, G., Ruff, J. P. C., Vaughey, J. T. & Osborn, R. (2020). *Nat. Mater.* **19**, 63–68. 10.1038/s41563-019-0500-7 31636421

[bb21] Roelsgaard, M., Dippel, A.-C., Borup, K. A., Nielsen, I. G., Broge, N. L. N., Röh, J. T., Gutowski, O. & Iversen, B. B. (2019). *IUCrJ*, **6**, 299–304. 10.1107/S2052252519001192 PMC6400190 30867927

[bb22] Shan, X., Guo, F., Page, K., Neuefeind, J. C., Ravel, B., Abeykoon, A. M. M., Kwon, G., Olds, D., Su, D. & Teng, X. (2019). *Chem. Mater.* **31**, 8774–8786.

[bb23] Simonov, A. (2019). *meerkat*. https://github.com/aglie/meerkat.

[bb24] Song, L., Roelsgaard, M., Blichfeld, A. B., Dippel, A.-C., Jensen, K. M. Ø., Zhang, J. & Iversen, B. B. (2021). *IUCrJ*, **8**, 444–454. 10.1107/S2052252521002852 PMC8086166 33953930

[bb25] Weber, T. & Simonov, A. (2012). *Z. Kristallogr.* **227**, 238–247.

[bb26] Yang, X., Masadeh, A. S., McBride, J. R., Božin, E. S., Rosenthal, S. J. & Billinge, S. J. L. (2013). *Phys. Chem. Chem. Phys.* **15**, 8480. 10.1039/c3cp00111c 23525376

